# Constitutional chromosomal events at 22q11 and 15q26 in a child with a pilocytic astrocytoma of the spinal cord

**DOI:** 10.1186/1755-8166-7-31

**Published:** 2014-05-15

**Authors:** Samantha Mascelli, Mariasavina Severino, Alessandro Raso, Paolo Nozza, Elisa Tassano, Giovanni Morana, Patrizia De Marco, Elisa Merello, Claudia Milanaccio, Marco Pavanello, Andrea Rossi, Armando Cama, Maria Luisa Garrè, Valeria Capra

**Affiliations:** 1 Istituto Giannina Gaslini, via G. Gaslini 5, 16147 Genoa, Italy

**Keywords:** Pilocytic astrocytoma, Spinal cord, Semicircular canal dysplasia, 15q duplication, 22q11.2 deletion

## Abstract

We report on a 9-years-old patient with mild intellectual disability, facial dimorphisms, bilateral semicircular canal dysplasia, periventricular nodular heterotopias, bilateral hippocampal malrotation and abnormal cerebellar foliation, who developed mild motor impairment and gait disorder due to a pilocytic astrocytoma of the spinal cord. Array-CGH analysis revealed two paternal inherited chromosomal events: a 484.3 Kb duplication on chromosome 15q26.3 and a 247 Kb deletion on 22q11.23. Further, a second *de novo* 1.5 Mb deletion on 22q11.21 occurred. Chromosome 22 at q11.2 and chromosome 15 at q24q26 are considered unstable regions subjected to copy number variations, i.e. structural alterations of genome, mediated by low copy repeat sequences or segmental duplications. The link between some structural CNVs, which compromise fundamental processes controlling DNA stability, and genomic disorders suggest a plausible scenario for cancer predisposition.

Evaluation of the genes at the breakpoints cannot account simultaneously for the phenotype and tumour development in this patient. The two paternal inherited CNVs arguably are not pathogenic and do not contribute to the clinical manifestations. Similarly, although the *de novo* large deletion at 22q11.21 overlaps with the Di George (DGS) critical region and results in haploinsufficiency of genes compromising critical processes for DNA stability, this case lacks several hallmarks of DGS.

## Background

Patients harbouring deletions within chromosome 22q11 present a wide array of clinical phenotypes, including DiGeorge, Velo-Cardio-Facial (DGS/VCFS) or 22q11.2 deletion syndrome [1-3]. Few reports of malignancy in patients with 22q11.2 deletion syndrome exist, including kidney tumours, hepatoblastomas, neuroblastomas, lymphomas, and, very rarely, central nervous system (CNS) tumours [4-7]. About 97% of DGS/VCFS patients have either a ~3 Mb deletion or, less commonly, a smaller ~1.5 Mb nested deletion [MIM 188400] [1]. Indeed, the long arm of chromosome 22 at band q11.2 is one of the most unstable regions with eight different specific low-copy repeats, designated LCR22s (LCR22-A-LCR22-H), which can yield recurrent Copy Number Variants (CNVs), such as microdeletions and microduplications [8]. A similar unstable region is present even on chromosome 15 at band q24q26 [9]. While the significance of CNVs to human pathology is being recognized, it is unclear how common or functionally relevant CNVs are to the process of carcinogenesis [10].

We present the clinical features of a 9-years-old male harbouring two paternally inherited events – a short 22q11.23 deletion and a 15q26.3 duplication – and a *de novo* 1.5 Mb deletion at 22q11.2 band. The child developed a spinal cord pilocytic astrocytoma (PA) [11-13]. We previously reported the cytogenetic characterization of the child and his family, suggesting that parental microdeletions/duplications could predispose to the formation of *de novo* pathogenically distinct CNVs in offspring [14]. Herein, we attempt to correlate the multiple CNVs with both development of the spinal cord PA and clinical phenotype.

## Case presentation

### Clinicopathological description

**Figure 1:**
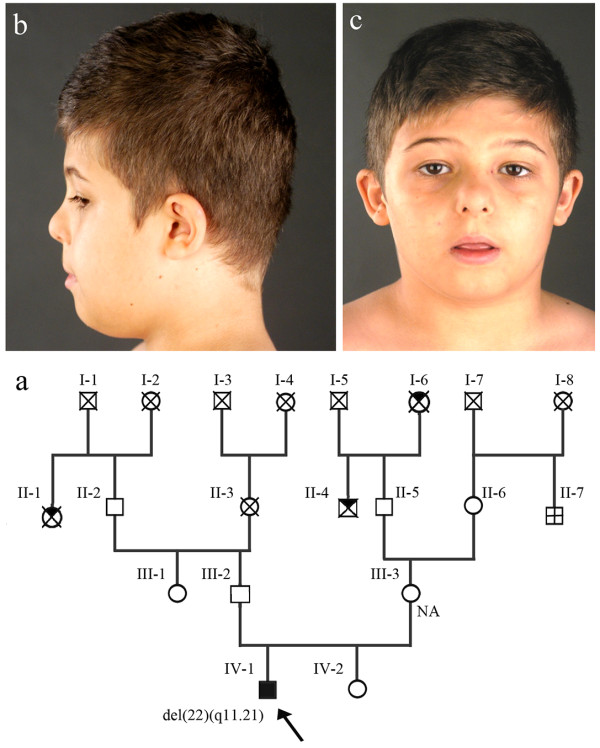
**Clinical features of the proband and family pedigree. (a)** In the family pedigree the propositus (IV-1) is indicated by a full-black square and an arrow - bold cross within square (II-7) indicates the second degree uncle with *pes cavus* - black corner of square (II-4) and circle (II-1) indicate second degree relatives who died for tumour under 55 years - black corner of circle (I-6) indicate a second degree aunt informative for the patient - NA indicates no chromosomal abnormalities. **(b, c)** The patient presented mild facial dimorphisms.

Patient’s clinical history, including information on family pedigree, and cytogenetic findings were previously reported (Figure 1) [14].

The child came to our attention at the age of 9 years, after a 3-year history of gait disturbance and right leg pain initially responding to anti-inflammatory therapy. Neurological examination revealed *pes cavus*, motor clumsiness, gait abnormalities with toe-walking and positive heel walk test. Somato-sensory evoked potentials showed low-amplitude potentials in lower limbs. Psychometrics confirmed mental retardation (Raven’s Matrices, PM47 = 20^th^ percentile). Electroencephalography was unremarkable. Mild obesity with increased abdominal fat distribution was present. Ophthalmologic examination was normal, while audiometric tests revealed left conductive hearing loss. Cardiac or urologic malformations were absent, as well as immune deficiency or congenital hypocalcaemia.

### Neuroimaging and pathological examination

**Figure 2:**
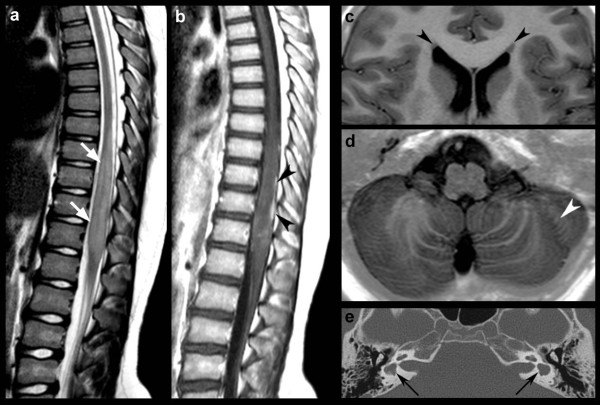
**Craniospinal MRI (a-d) and temporal bone CT (e) images.** Spine MRI reveals a solid tumour mass hyper intense on sagittal T2-weighted images (**a**, white arrows) with mild and inhomogeneous contrast enhancement on Gd-enhanced sagittal T1-weighted images (**b**, black arrowheads). Brain MRI axial STIR show bilateral frontal periventricular nodules of gray matter heterotopias (**c**, black arrowheads) and an area of abnormal cerebella foliation (**d**, white arrowhead). Temporal bone CT scan depicts bilateral digenesis of the lateral semicircular canals and vestibules forming a single cavity with normal appearing cochleae (**e**, black arrows).

Spine magnetic resonance imaging (MRI) revealed enlargement of the inferior dorsal spinal cord due to a solid tumour, hypointense on T1-weighted images and hyperintense on T2-weighted images, with mild, inhomogeneous contrast enhancement. Leptomeningeal invasion and dissemation were absent (Figure 2a, b). Brain MRI showed small nodules of subependymal gray matter heterotopia, bilateral hippocampal malrotation, and abnormal cerebellar foliation (Figure 2c, d). Temporal bone computerized tomography revealed bilateral digenesis of the lateral semicircular canals and vestibules forming a single cavity with normal appearing cochleae (Figure 2e). Signs of left chronic otitis media were found.

After a period of surveillance, the patient underwent neurosurgical intervention. The absence of a cleavage plane between the tumour and the surrounding tissue hampered total or subtotal resection, due to high risk of functional damage. Therefore, only a biopsy was performed. Histological findings were consistent with PA. The labelling index, as evaluated by the monoclonal antibody Ki67-MIB-1, was 1%. No expression of p53 protein was found.

Adjuvant therapies were planned to be introduced just in case of clinical and/or imaging worsening. After four years, the child is neurologically stable with no remarkable change in tumour volume.

### CNV analysis

CNVs were analyzed using UCSC Genome Browser (http://www.genome.ucsc.edu) and Decipher Database (http://decipher.sanger.ac.uk/, version 6.0) website tools. The 15q26.3 duplication, with proximal breakpoints between base pairs 100,051,182 and 100,996,155, involved myocyte enhancer factor 2A (*MEF2A*) [MIM 600660], a target of ERK/MAPK pathway. Defects in this gene were reported in autosomal dominant coronary artery disease 1 with myocardial infarction (*ADCAD1*) [MIM 608320]. The duplication also involved putative peptidoglycan-binding domain containing 4 (*LYSMD4*) [XM_005254863], ADAM metallopeptidase with thrombospondin type 1 motif, 17 (*ADAMTS17*) [DQ_217943] and interrupted ceramide synthase 3 (*CERS3*) [MIM 615276]. Mutations in *ADAMTS17* can cause autosomal recessive Weill-Marchesani-like syndrome [MIM 613195] while alteration of both *ADAMTS17* and *CERS3* have been associated with autosomal recessive congenital ichthyosis 9 (ARCI9) [MIM 615023].

The 22q11.23 deletion (chr22:25,664,618-25,911,651), involving low density lipoprotein receptor-related protein 5-like (*LRP5L*) [NM_182492], has never been associated with any disease.

The *de novo* 22q11.21 deletion (chr22:18,894,835-20,311,763) involved 24 genes located within the DGS critical region (*DGCR*). Besides T-box 1 (*TBX1*), a transcription factor regulating developmental processes, several genes are involved, as clathrin, heavy chain-like 1 (*CLTCL1*) [MIM 601273], RAN binding protein 1 (*RANBP1*) [MIM 601180], histone cell cycle regulator (*HIRA*) [MIM 600237], cell division cycle 45 (*CDC45*) [MIM 603465], and catechol-O-methyltransferase (*COMT*) [MIM 116790] partially overlapping thioredoxin reductase 2 (*TXNRD2*) [MIM 606448]. These genes are active in many cellular processes, including cell cycle progression, senescence, signal transduction, apoptosis and gene regulation.

## Discussion

Locus-specific rates of CNV formation underlie not just rare complex disorders involving multiple congenital abnormalities but also sporadic diseases, and perhaps cancer predisposition. The principal models currently invoked to explain genomic instability and susceptibility to cancer, conjecture the deleterious consequences of some structural CNVs on fundamental processes, including DNA replication and damage response (DDR) processes [8,15].

The child paternally inherited the 15q26.3 duplication. This distal duplication involved four genes active in several cellular processes encompassing *MEF2A* which is a target of ERK/MAPK pathway. Although the pathway plays an important role in PA [16], defects in *MEF2A* have never been associated to this tumour. On the other hand, paternally inherited 22q11.23 short deletion arguably contribute neither to clinical phenotype nor to tumour development.

The present case had a *de novo* 1.5 Mb deletion at chromosome 22q11.21 (Figure 1a), one of the most rearrangement-prone regions in the human genome associated with genetic disorders including DGS/VCFS.

Breakpoints analysis revealed that the heterozygous deletion contains several genes of the critical region for DGS/VCFS, i.e. several DGCRs, encompassing *TBX1*, who is responsible for most of malformations of this syndrome [17]. Twenty-four genes resulted haploinsufficient including cell division cycle 45 (*CDC45*), catechol-O-methyltransferase (*COMT*) and thioredoxin reductase 2 (*TXNRD2*) which could increase risk of malignancy in patients with 22q11.2 DS [4,8,18].

Although 22q11.21 deletion overlaps with the DGS/VCFS region [2], the clinical phenotype was only partially related with this syndrome making untangling genotype-phenotype relationship difficult. In fact, several hallmarks of 22q11 deletion syndrome lacked, such as palatal abnormalities, immuno-deficiency, cardiac and genitourinary malformations, and hypocalcaemia [2,3]. However, the patient presented motor and language delay with behavioural immaturity consistent with the DGS/VCFS “neurobehavioral” phenotype [18,19]. The child additionally had periventricular gray matter heterotopias, hippocampal malrotation and cerebellar foliation abnormalities. Brain malformations are frequent in DGS/VCFS patients, including polymicrogyria, callosal agenesis and cerebellar vermian hypoplasia (Figure 2c, d) [20]. Intriguingly, bilateral inner ear malformations were found, consisting in bilateral semicircular canal dysplasia (Figure 2e). This abnormality has been occasionally described in DGS/VCFS [21], but its clinical significance is unknown due to the lack of correlation with sensor-neural hearing loss.

This child developed a spinal cord PA (WHO grade I) at the age of 9 years. This rare neoplasm accounts for 2-5% of all CNS tumours in children and young adults [12,13]. PA may be encountered in tumour syndromes like Neurofibromatosis type 1 [22] and, rarely, Frasier and Noonan syndromes [23,24].

Only sporadic cases of brain tumour in children with 22q11 deletion syndrome were reported [5-7]. Indeed, cytogenetic studies demonstrated the association between CNS rhabdoid tumors and chromosome 22q11.2 alterations, with *SMARCB1* [MIM 601607] deletions and/or mutations [7], while the first case of pleomorphic xanthoastrocytoma in a 16 year-old patient with DGS/VCFS, harbouring V600E *BRAF* mutation has been recently reported [6]. The identified 22q11.2 deletion was proximal and did not encompass *SMARCB1* locus.

The combination of 22q11.1 deletion with a proximal 15q11.2-q13.3 duplication was described, in a case with psychomotor delay, mild facial dysmorphisms, learning difficulties, but without tumour [25].

This spinal PA likely represent a sporadic event and relationship with DGS/VCFS is uncertain. Thus, despite extent of the *de novo* deletion at 22q11.2 and little contribution of the paternal inherited CNVs, no features apparently correlated with either tumour development or phenotypic defects. Accordingly, the possibility of the occurrence of the spinal tumour merely by chance should be considered.

## Conclusion

Paternal inherited CNVs did not lead to pathological consequences. Even no consistent genotype-phenotype correlation has been shown for the 22q11.21 deletion: a definite link between tumour and chromosomal event does not exist. Perhaps the *de novo*, large 22q11.21 deletion, apparently together with the inherited benign variants, may contribute to development of both spinal PA and complex phenotype.

These novel observations warrant ongoing reporting of similar cases to untangle the complex genotype-phenotype relationship with constitutional 22q11.2 deletion. The biological consequences and the contribution to cancer predisposition of chromosomal rearrangements involving CNVs of some DDR components deserve in-depth investigation.

## Consent

Written informed consent was obtained from the patient’s parents for publication and any accompanying images of this case report. A copy of the written consent is available for review by the Editor-in-Chief of this journal.

## Abbreviations

DGS/VCFS: DiGeorge or Velo-cardio-facial syndrome; CNS: Central nervous system; PA: pilocytic astrocytoma; MRI: Magnetic resonance imaging; WHO: World Health Organization; CNV: Copy number variation; DDR: DNA damage response; ADCAD1: Autosomal dominant coronary artery disease 1 with myocardial infarction; ERK/MAPK: Extracellular signal-regulated kinases/ Mitogen-activated protein kinases; MEF2A: Myocyte enhancer factor 2A; LYSMD4: putative peptidoglycan-binding, domain containing 4; ADAMTS17: ADAM metallopeptidase with thrombospondin type 1 motif, 17; CERS3: Ceramide synthase 3; ARCI: Autosomal recessive congenital ichthyosis; LRP5L: Low density lipoprotein receptor-related protein 5-like; DGCR: DiGeorge syndrome critical region genes; TBX1: T-box 1; CLTCL1: Clathrin, heavy chain-like 1; HIRA: Histone cell cycle regulator; RANBP1: RAN binding protein 1; CDC45: Cell division cycle 45; COMT: Catechol-O-methyltransferase; TXNRD2: Thioredoxin reductase 2.

## Competing interests

The authors declare that they have no competing interests.

## Authors’ contributions

SM and MS provided assistance for the clinical data, participated in the design of the study and wrote the manuscript. AR participated in coordination of the study. GM, CM, AR and PN carried out radiological and pathological data review, respectively, and gave editorial assistance. ET carried out cytogenetic studies. PDM revised the manuscript and with EM and MP gave editorial assistance. AC provided clinical data. MLG participated in the design of the study, provided clinical data and helped to draft the manuscript. VC conceived the study, participated in its design and coordination and drafted the manuscript. All authors read and approved the final manuscript.
